# Understanding nitrate assimilation and its regulation in microalgae

**DOI:** 10.3389/fpls.2015.00899

**Published:** 2015-10-26

**Authors:** Emanuel Sanz-Luque, Alejandro Chamizo-Ampudia, Angel Llamas, Aurora Galvan, Emilio Fernandez

**Affiliations:** Department of Biochemistry and Molecular Biology, University of CordobaCordoba, Spain

**Keywords:** nitrate assimilation, nitrate/nitrite uptake, green algae, nitric oxide, nitrogen metabolism, *Chlamydomonas*

## Abstract

Nitrate assimilation is a key process for nitrogen (N) acquisition in green microalgae. Among Chlorophyte algae, *Chlamydomonas reinhardtii* has resulted to be a good model system to unravel important facts of this process, and has provided important insights for agriculturally relevant plants. In this work, the recent findings on nitrate transport, nitrate reduction and the regulation of nitrate assimilation are presented in this and several other algae. Latest data have shown nitric oxide (NO) as an important signal molecule in the transcriptional and posttranslational regulation of nitrate reductase and inorganic N transport. Participation of regulatory genes and proteins in positive and negative signaling of the pathway and the mechanisms involved in the regulation of nitrate assimilation, as well as those involved in Molybdenum cofactor synthesis required to nitrate assimilation, are critically reviewed.

## Introduction

The macroelement Nitrogen (N) is an essential component in key molecules of living mater. However, dinitrogen, the most abundant form either in the atmosphere or dissolved in waters, can only be used by fixing bacteria but is inaccessible to eukaryotic algae in general. Nevertheless, an uncommon symbiotic process between a unicellular cyanobacteria and a single-celled eukaryotic alga was described (Thompson et al., 2012). N can be incorporated in eukaryotic organisms from either organic or inorganic forms. The availability and concentrations of inorganic N sources, which in general are small, change depending on environments (Giordano and Raven, 2014) and usually limit growth and productivity. ${\text{NH}}_{4}^{+}$, ${\text{NO}}_{2}^{-}$, and ${\text{NO}}_{3}^{-}$ the most frequent inorganic N sources assimilated by photosynthetic organisms show a different spatial distribution in oceans. In the euphotic zone of ocean waters (the area closer to the surface, which receives enough light for photosynthesis to be carried out), the estimated mean concentration of nitrate is about 7 μM being those of ammonium and nitrite much smaller, 0.3 and 0.1, respectively. Whereas in the aphotic zone (just below the euphotic zone and unable to support photosynthetic or autotrophic growth) these values are 31, 0.01, 0.006 for nitrate, ammonium, and nitrite, respectively (Gruber, 2008). However, in the soil nitrate concentrations can be very variable from 10 μM to 100 mM (Crawford, 1995). In natural surface waters, nitrate concentration is usually < 1 μM, but it can increase several orders of magnitude specifically in underground waters mainly due to contamination from plant fertilizers or animal farms. Depending on colonized environments, microalgae own different adaptations for a proper N assimilation.

### Interest of using *Chlamydomonas* in nitrate assimilation studies

In this review, we will focus on nitrate assimilation in microalgae, which has generated basic knowledge and contributed significantly to the understanding of the pathway in crop plants (Ho and Tsay, 2010; Chardin et al., 2014; Krapp et al., 2014). Algae are a group of polyphyletic organisms, which evolved along different endosymbiotic events, thus the several species developed different and distinctive cell structures, grouping organizations, and metabolisms. Here, we will only refer to algae whose ancestors derive from a primary endosymbiotic event (de Clerck et al., 2012). They correspond to individuals from the group of Glaucocystophytes, Rhodophytes, and Chlorophytes. This latter group of chlorophytes is phylogenetically closely related to plants. Other algae groups add new layers of complexity, since they come from secondary and tertiary endosymbiotic events, with plastid loss, replacement, and gene transfers (Douglas, 1998; Keeling, 2010). Among those algae *Chlamydomonas reinhardtii* (referred herein as *Chlamydomonas*) has emerged as a very relevant experimental system, mostly due to the development of molecular and genetic tools useful in many different studies, such as the flagella structure and function, photosynthetic apparatus, chloroplast evolution, metabolism, or regulation under different stress conditions (Harris, 2009). Advances achieved in understanding of nitrate assimilation in this system (Fernandez and Galvan, 2007, 2008) were extrapolated to other algae and mostly to plants leading to remarkable progress (Xu et al., 2012; Bittner, 2014; Krapp et al., 2014). Many of the methodological tools in *Chlamydomonas* rely on its efficient transformation system, development of vectors (Kindle, 1990; León-Bañares et al., 2004; Neupert et al., 2012) and the sequencing of its genome (Merchant et al., 2007). In other algae, transformation methodology has been set up (Sun et al., 2006; Lerche and Hallmann, 2009, 2013, 2014; Hirata et al., 2011; Qin et al., 2012; Rathod et al., 2013; Talebi et al., 2013; Yamano et al., 2013), which will allow efficient techniques to appear as in *Chlamydomonas*. We will refer here to 10 Chlorophytes (using *Chlamydomonas* as a reference), two Rhodophytes, and a Glaucophyte, for which their genomes are sequenced and available in the public databases.

## Nitrate assimilation

### Overview of nitrate assimilation

The nitrate assimilation pathway from nitrate to amino acid is relatively simple at structural level. By contrast, its regulation to ensure the efficient assimilation of nitrate coupled to that of other environmental factors, is complex.

#### Overview in chlamydomonas/algae

In photosynthetic eukaryotes, nitrate assimilation is performed by two transport and two reduction steps: First, nitrate is transported into the cell, then a cytosolic Nitrate Reductase (NR) catalyzes nitrate reduction to nitrite, which subsequently is transported into the chloroplast, where the enzyme Nitrite Reductase (NiR) catalyzes its reduction to ammonium (Guerrero et al., 1981; Fernandez and Galvan, 2007, 2008). Finally, ammonium is incorporated to carbon skeletons by rendering glutamate, through the glutamine synthetase/glutamine oxoglutarate amino transferase or glutamate synthase (GS/GOGAT) cycle (Miflin and Lea, 1975). First, ammonium is incorporated as the amide group of glutamine in a reaction involving glutamate and ATP (catalyzed by GS); then, the amide group is transferred reductively to α-oxoglutarate to form two molecules of glutamate.

**Figure 1:**
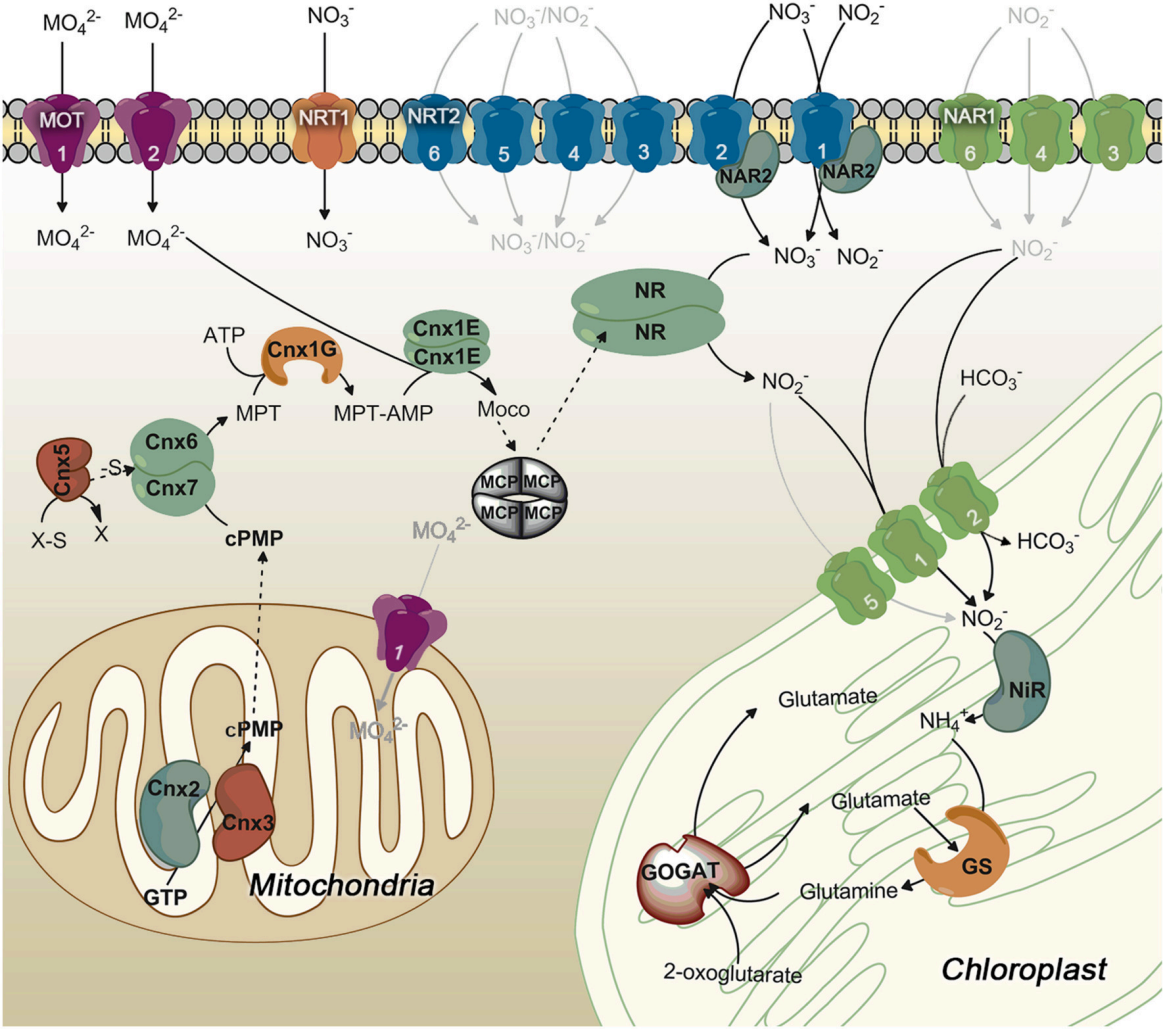
**General scheme of proteins involved in nitrate assimilation and molybdenum cofactor biosynthesis in *Chlamydomonas***. Those steps symbolized in gray lines are not empirically demonstrated. Proteins that mediate these hypothetic steps are represented in the most probable localization. Different colors are used for each family of transporters. Numbers on the transporters identify each family member.

Figure 1 summarizes the organization of enzymes/proteins for nitrate assimilation in *Chlamydomonas*. The assimilation of nitrate begins with its entry into the cell. The transport of nitrate and nitrite into the cell is a regulated and complex process in *Chlamydomonas* as suggested from the high number and types of proteins that participate (Fernandez and Galvan, 2007). The transporters are classified, based on their substrate specificity, affinity and expression characteristics, in: specific or bispecific, high and low affinity, constitutive and inducible transporters. From the structural point of view three families of proteins are involved in nitrate and/or nitrite transport in *Chlamydomonas*. They are NRT1 (NPF), NRT2, and NAR1 (Crawford and Glass, 1998; Forde, 2000; Galvan and Fernández, 2001; Forde and Cole, 2003; Fernandez and Galvan, 2007, 2008).

#### NRT1 transporters function

The NRT1 transporters belong to the PTR transporters (Peptide TRansporters), now renamed NPF for NRT1/PTR Family (Léran et al., 2014). There is a wide representation of these transporters in plant genomes (Krapp et al., 2014), however its presence in algal genomes is in low number if any. Most of the plant NRT1 genes encode low-affinity nitrate transporters (Tsay et al., 2007). The best-studied one is CHL1 from *Arabidopsis* (AtNRT1.1 or AtNPF2.6) and represents a paradigmatic protein. In fact, AtNRT1.1 is considered a transceptor with sensor and carrier functions (Tsay et al., 2007; Ho et al., 2009; Gojon et al., 2011). AtNRT1.1 is a dual affinity nitrate transporter that switches from high to low-affinity for nitrate, depending on the nitrate conditions and the phosphorylation/dephosphorylation of the threonine T101. At low nitrate conditions, AtNRT1.1 is phosphorylated by a CIPK23 protein kinase and functions as a high-affinity transporter. At high nitrate conditions AtNRT1.1 is dephosphorylated and is a low-affinity transporter (Liu et al., 1999; Ho et al., 2009). In addition AtNRT1.1 is involved in plant auxin transport and nitrate efflux (Tsay et al., 2007; Ho et al., 2009; Gojon et al., 2011).

#### NRT2 transporters function

The NRT2 proteins correspond to the NNP (Nitrate Nitrite Porter) family and belongs to the superfamily MFS (Major Facilitator Superfamily; Pao et al., 1998). *NRT2* genes are present into genomes of organisms able to assimilate nitrate like plants, algae, fungi, yeast and bacteria (Forde, 2000). The NRT2 proteins can transport nitrate or nitrite and some of them are two-component systems, which require a second protein, NAR2, to be fully functional. NAR2 appears to be anchored to the membrane by a transmembrane domain (Quesada and Fernández, 1994; Tong et al., 2004; Okamoto et al., 2006; Orsel et al., 2006). In *Chlamydomonas* there are six NRT2 members but NAR2 is only required for functionality of NRT2.1 and NRT2.2 but not for other NRT2 transporters (Quesada and Fernández, 1994; Galván et al., 1996). Thus, in *Chlamydomonas* NRT2.1/NAR2 constitutes a bispecific high-affinity nitrate/nitrite transporter and NRT2.2/NAR2 is a high-affinity nitrate transporter (Fernandez and Galvan, 2007).

#### NAR1 transporters function

The family NAR1 (Nitrate Assimilation-Related component 1) is formed by transporters related to proteins called FNT (Formate Nitrite Transporters; Peakman et al., 1990; Suppmann and Sawers, 1994; Rexach et al., 2000). *Chlamydomonas* has six *NAR1* genes (*NAR1.1–6*; Mariscal et al., 2006). NAR1.1 is a nitrite transporter to the chloroplast (Rexach et al., 2000), and NAR1.2 (LCIA) shows ${\text{NO}}_{2}^{-}$ and ${\text{HCO}}_{3}^{-}$ transport activity when expressed in *Xenopus* oocytes (Mariscal et al., 2006). More recently, it has been demonstrated that NAR1.2 (LCIA) is a chloroplast envelope ${\text{HCO}}_{3}^{-}$ transporter and part of a CO_2_-concentration mechanism (CCM) operating at very low CO_2_ (Wang and Spalding, 2014; Yamano et al., 2015). The CCM is essential to accumulate CO_2_ close to RUBISCO and make efficient photosynthesis in aquatic microalgae.

#### Nitrate reductase function

The enzyme NR catalyzes the reduction of nitrate to nitrite with electrons from NAD(P)H. In eukaryotes, NR is a homodimeric protein with subunits of about 100–120 kDa containing each one three prosthetic groups: FAD, b_557_ heme and Molybdenum cofactor (Moco) (Zhou and Kleinhofs, 1996). Algae typically contain a single gene encoding NR (Fernández et al., 1989; Fernandez and Galvan, 2007) that, in addition to its physiological activity of nitrate reduction, has also two other activities. These partial activities can be assayed separately *in vitro*. The first one is the NAD(P)H-dehydrogenase or diaphorase, which catalyzes the transfer of electrons from pyridine nucleotides to artificial electron acceptors such as ferricyanide, dichlorophenol indophenol, or cytochrome c. This activity does not require the Moco domain. The second is the terminal activity, which catalyzes the transfer of electrons from artificial electron donors as flavins, bromophenol blue, or viologens (chemically reduced with dithionite) to nitrate (Solomonson et al., 1986; Caboche and Rouzé, 1990). This activity mostly requires the Moco domain.

#### Nitrite reductase function

Nitrite reduction to ammonium occurs in the stroma of chloroplasts and is catalyzed by the enzyme nitrite reductase (NiR). Reduced ferredoxin (Fd_red_) generated as a consequence of the photosynthetic electron transport is the electron donor for nitrite reduction. In the dark, nitrite reduction can also occur from NADPH, generated in the oxidative pentose phosphate cycle, by mediation of Fd-NADP^+^ oxidoreductase (Jin et al., 1998). Algal NiR, like in cyanobacteria and plants, is a monomer of approximately 63 kDa containing a [4Fe–4S] grouping and a siroheme as prosthetic groups. NiR is encoded by a single gene in *Chlamydomonas*, which is clustered with other genes essential for nitrate/nitrite assimilation (Guerrero et al., 1981; López-Ruiz et al., 1991; Quesada et al., 1993).

#### Incorporation of N into amino acids

Inorganic N is incorporated finally in form of organic N as glutamate by the action of GS/GOGAT (see above). *Chlamydomonas* contains four GS (GLN) (two cytosolic and two plastid-targeted), and two plastidic GOGAT, one dependent on NADH and the other on Fd _red_ (Cullimore, 1981; Cullimore and Sims, 1981a,b; Fischer and Klein, 1988; Chen and Silflow, 1996). GLN1 and GLN2 from *Chlamydomonas* have high sequence conservation and a similar octameric structure (Florencio and Vega, 1983a; Chen and Silflow, 1996). Chen and Silflow (1996) found three forms of GS in *Chlamydomonas*, but the sequencing of its genome detected four (Merchant et al., 2007). However, function and localization of these two additional GS (GLN3 and GLN4) have not been studied in detail.

In addition to GS/GOGAT, under certain stress conditions glutamate dehydrogenase (GDH) seems to be the predominant enzyme for ammonium incorporation in *Chlamydomonas*, catalyzing a reductive reaction of ammonium and α-oxoglutarate to yield glutamate (Muñoz-Blanco and Cárdenas, 1989). GDH is encoded by two genes (*GDH1* and *GDH2*) and has three isoenzymes located in the mitochondria, resulting very probably from combination of the two different subunits (Moyano et al., 1992; Vallon and Spalding, 2009). Under stress conditions such as N-starvation or high ammonium, the aminating activity of GDH varies inversely to that of GS. The GDH function seems to be related to the maintenance on glutamate levels in the cell when GS/GOGAT is inefficient, appearing to be more connected to carbon than to nitrogen metabolism (Muñoz-Blanco and Cárdenas, 1989; Moyano et al., 1995). Since GDH is related to ammonium assimilation, which is not the topic of this review, it will not be mentioned any further.

### Genes for nitrate assimilation in algae

Table 1 (more details in Table S1) shows the genes and number of genes involved in nitrate assimilation in several Chlorophytes, Rhodophytes, and Glaucophytes algae with sequenced genomes.

**Table 1:** **Nitrate and molybdenum assimilatory proteins in microalgae**.

	***Chlorophytes***										***Rhodophytes***		***Glaucophytes***
	***C. reinhardtii***	***V. carteri***	***C. subellipsoidea***	***M. pusilla***	***Micromonas RCC299***	***Chlorella NC64A***	***O. lucimarinus***	***Ostreococcus RCC809***	***O. tauri***	***B. prasinos***	***C. merolae***	***G. sulphuraria***	***C. paradoxa***
NRT1	1	1	2	NF	1	2	1	1	1	1	1	1	NF
NRT2	6	3	1	1	3	2	1	1	1	1	1	1	2
NAR1	6	5	2	1	1	3	1	1	1	1	NF	NF	NF
NAR2	1	1	1	1	2	1	1	1	1	1	NF	NF	NF
NR	1	1	2	1	1	1	1	1	1	1	1	1	1
NiR	1	1	1	1	1	1	1	1	1	1	2	1	1
GLN	4	4	3	1	1	2	1	1	1	1	1	1	2
GSN/GSF	2	2	2	2	2	2	1	1	1	1	1	1	1
MCP	1	1	NF	NF	NF	NF	NF	NF	NF	NF	NF	NF	NF
MOT1	1	NF	1	NF	1	1	1	1	1	1	NF	NF	2
MOT2	2	2	2	2	2	2	2	2	2	2	1	1	1
CNX2	1	1	1	1	1	1	1	1	1	1	1	1	1
CNX3	1	1	1	1	1	1	2	1	1	1	1	1	1
CNX5	1	1	1	1	1	1	1	1	1	1	1	1	1
CNX6	1	1	1	1	1	1	1	1	1	1	1	1	1
CNX7	1	1	1	1	1	1	1	1	1	1	NF	NF	NF
CNX1G	1	1	1	1	1	1	1	1^*^	1^*^	1	1	1	NF
CNX1E	1	1	1	1	1	1	1	1^*^	1^*^	1	1	1	1
ABA3	1	1	1	NF	1	1	NF	NF	NF	1	NF	NF	NF

We have used the specific genome databases for each organism. Some proteins were already identified and annotated and others were found using BlastP and TBlastN. Chlamydomonas reinhardtii: Phytozome 10.2 (http://phytozome.jgi.doe.gov/pz/portal.html), for NAR1(192094) we used JGI v4 (http://genome.jgi-psf.org/). Volvox carteri, Coccomyxa subellipsoidea, Micromonas pusilla, Micromonas RCC299 and Ostreococcus lucimarinus: Phytozome 10.2. Chlorella NC64A, Ostreococcus RCC809 and Ostreococcus tauri: JGI. Bathycoccus prasinos: Genome.jp (www.genome.jp/kegg-bin/show_organism?org=bpg). Cyanidioschyzon merolae: Cyanidioschyzon merolae Genome Project (http://merolae.biol.s.u-tokyo.ac.jp/blast/blast.html). Galdieria Sulphuraria: (http://genomics.msu.edu/cgi-bin/galdieria/blast.cgi). Cyanophora paradoxa: (http://cyanophora.rutgers.edu/cyanophora/blast.php). Identified proteins are NRT1, NRT2, and NAR1, nitrate/nitrite transporters; NAR2, required component for high affinity nitrate transport; NR, Nitrate Reductase; NiR, Nitrite Reductase; GLN, Glutamine synthetase; GSN/GSF, Glutamate synthase; MCP, a Moco Carrier Protein; MOT1 and MOT2, Molybdate Transporters; Cnx2, Cnx3, Cnx5, Cnx6, Cnx7, Cnx1G, Cnx1E, and ABA3, Proteins of Molybdenum cofactor biosynthesis; Accession numbers of all these proteins are provided in Table S1. In Table S1 those proteins encoded by clustered genes are also indicated. (NF) indicates that proteins were not found in that organism. The asterisk (^*^) indicates that CNX1G and CNX1E are fused into a single chimeric gene in those algal species.

The *NRT1 (NPF)* genes are represented in high number in plant genomes. In contrast, the algal genomes contain a very low number, two, one, or even none. For example, *Arabidopsis* has 53 NRT1 (NPF) but the number in plants varies between 51 (in *Capsella rubella*) and 139 (in *Malus domestica*; Crawford and Glass, 1998; Williams and Miller, 2001; Léran et al., 2014). *Chlamydomonas* has a unique *NRT1*, like most of the algal genomes analyzed. *Coccomyxa subellipsoidea* and *Chlorella NC64A* contain two, but the *NRT1* gene was not found in the microalgae *Micromonas pusilla* or *Chlorella paradoxa* (Table 1). This is relevant since, provided that the analyzed genomes are complete, it suggests that along evolution some important function mediated by NRT1 has been maintained in the different algae species, and that in plants, the complexity of the new tissues that were appearing required new functions, which were assumed by this family of proteins.

The NRT2 proteins, responsible for high affinity nitrate/nitrite transport (HATS), are present in all algal species. However, NAR2, which with NRT2 forms the two-components HATS appears in Chlorophytes algae, like in plants, but not in Rhodophytes or Glaucophytes (Table 1), suggesting that in these latter algae one-component nitrate HATS might be efficient enough to guarantee cell growth and performance. NAR2 is highly conserved between *Chlamydomonas* and *Volvox* but show a lower conservation with the other Chlorophytes. So far, *Chlamydomonas* is the alga with the highest number of *NRT2* genes, likewise for *NAR1* genes (six of each). Like *NAR2, NAR1* was not found in the genomes of Rhodophytes or Glaucophytes, and the number of *NAR1* genes is just one in species of *Micromonas, Ostreococcus*, and *Bathycoccus prasinos*. This is interesting because in *Chlamydomonas* two members of this NAR1 family have specialized functions. NAR1.1 is a nitrite transporter to the chloroplast clustered with the nitrate assimilation genes. The gene encoding this transporter is expressed in the presence of nitrate under the control of *NIT2*, which is the major regulatory gene for nitrate assimilation. All this suggests a role of NAR1.1 in nitrate assimilation (Fernandez and Galvan, 2007, 2008). On the other hand, *NAR1.2* (*LCIA*) is not nitrate-regulated nor *NIT2*-dependent (Mariscal et al., 2006). NAR1.2 is expressed under low CO_2_ conditions and is a ${\text{HCO}}_{3}^{-}$ transporter to the chloroplast (Wang and Spalding, 2014; Yamano et al., 2015). The function of CrNAR1.3–6, as that of the single NAR1 in other microalgae species, has to be solved. The high number of these genes, NRT2 and NAR1, in *Chlamydomonas* might reflect an optimization in the utilization of those nutrients under many diverse environmental conditions.

Single genes encode NR and NiR in algae, excepting some particular cases as *Coccomyxa subellipsoidea* C-169, which has two NRs in its genome (Table 1). It is interesting to point out that in *Cyanidioschyzon merolae*, which lacks NiR gene (*NII1*), one sulfite reductase gene *CmSiRB* of the two annotated in its genome, *CmSiRA* and *CmSiRB*, encodes really a nitrite reductase (Sekine et al., 2009). Sulfite reductase and nitrite reductase have common structural and functional features (Crane and Getzoff, 1996). The nitrite reduction function of CmSiRB was deduced from its high NiR vs. its very low sulfite reductase activity, high expression in nitrate medium, and low in ammonium medium in contrast to *CmSiRA*, which is not affected by the presence of ammonium or nitrate. In addition, in the genome *CmSiRB* maps between a nitrate transporter and the NR genes (Sekine et al., 2009).

**Figure 2:**
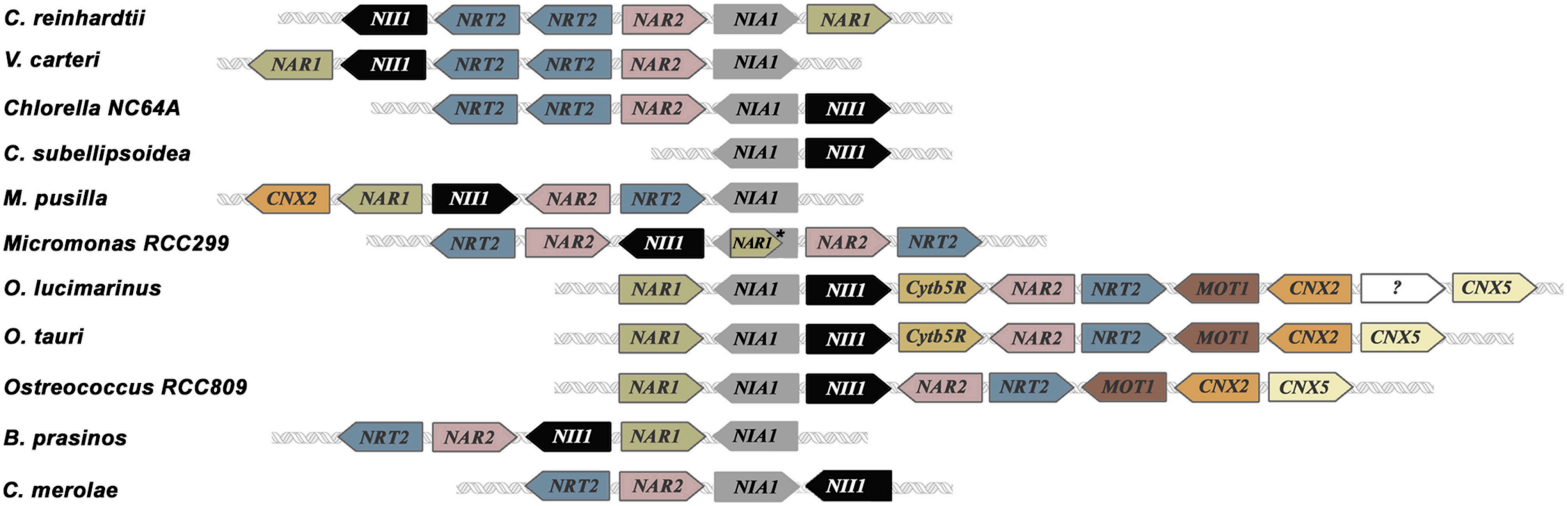
**Clusters of nitrate assimilation genes in microalgae**. *NIA1* and *NII1* (genes encoding NR and NiR) are the only genes conserved in all clusters represented. All clusters have been arranged to show *NIA1* gene fixed in the middle of the figure. All the genes represented are involved in nitrate assimilation and molybdenum metabolism with exception of Cytb5R whose relationship with nitrate metabolism is unknown. ^*^In *Micromonas* RCC299 *NIA1* and *NAR1* are overlapped.

Clustering of nitrate assimilation genes appears commonly in fungi, cyanobacteria and algae (Johnstone et al., 1990; Frías et al., 1997; Fernandez and Galvan, 2008; McDonald et al., 2010; Maruyama and Archibald, 2012). In fungi the metabolic gene clusters are often associated with fungal virulence, and evolutionarily with pathways conferring some advantage over competitors. They might have appeared by horizontal gene transfer (Slot and Hibbett, 2007; Wisecaver and Rokas, 2015). In plants, metabolic gene clusters are more common than suspected since the seminal work of Osbourn et al. (2012). However, the reasons for this clustering are not yet understood, but it is probable that this physical proximity of genes facilitates the coordination of their regulation and inheritance (Osbourn et al., 2012; Mugford et al., 2013; Nützmann and Osbourn, 2015). The clustering of nitrate assimilation genes in algae is analyzed in Table S1 and Figure 2. Though the particular genes clustered change in its role, order or orientation from species to species, it is interesting to point out that most of Chlorophytes have a complete set of genes for nitrate assimilation (*NRT2/NAR2/NIA1/NII1/NAR1*) with the exception of *Chlorella sp. NC64A*, which has three *NAR1* but none within the nitrate-cluster and *Cocomyxa subellipsoidea* in which genes are scattered in the genome. A curiosity is that *Chlorella* sp. NC64A, even having a complete set of all the genes needed for nitrate assimilation (transporters, reductases and Moco genes), has developed an alternative adaptation strategy to acquire N. This *Chlorella* strain is endosymbiotic with *Paramecium bursaria* and can use ammonium or amino acids but not nitrate or nitrite (Kamako et al., 2005). In this alga NiR and not NR activity is considerably stimulated by nitrate but the mechanisms responsible for this silencing of nitrate utilization are unknown.

Another curiosity is that *Ostreococcus* species contain in addition to genes for nitrate/nitrite transporters and reductases others for molybdate transport (*MOT1*) and metabolism (*CNX2* and *CNX5*). Thus, the complete cluster in *Ostreococcus tauri* is: *NAR1-NIA1-NII1-Cytb5R-NAR2-NRT2-MOT1*-*CNX2-CNX5* (Derelle et al., 2006). In *Chlamydomonas* two gene clusters are present: one consists on *NII1-NRT2.2-NRT2.1-NAR2-NIA1-NAR1* and in the other *NRT2.3-AOX1* (*AOX1* encodes a mitochondrial alternative oxidase) (Quesada et al., 1998a,b). In *Micromonas* species two different clusters were described: *CNX2-NAR1-NII1-NAR2-NRT2-NIA1* (in *M. pusilla* CCMP1545), and *NRT2.1-NAR2.1-NII1-NAR1-NIA1-NAR2.2-NRT2.2* (in RCC299) (McDonald et al., 2010). A comparison between the different gene clusters for nitrate assimilation in microalgae is shown in Figure 2. It is remarkable how essential genes for nitrate assimilation have been maintained clustered during the different genetic events needed to configure the present organization.

Molybdate transporter genes (MOT), are present in the algal genomes highlighting the importance of this anion for a proper assimilation of nitrate (Table 1). Accordingly, the complete set of genes for Moco biosynthesis is represented in all the species analyzed. Interestingly, the *CNX1* gene, encoding the two domains (E and G) protein in plants, is split into two genes each one encoding a domain, *CNX1G* and *CNX1E*. However, some exceptions are found in *Ostreococcus tauri, Ostreococcus RCC809*, and *Bathycoccus prasinos* where these domains are merged in a single gene. By fusing those two genes from *Chlamydomonas reinhardtii* in chimeric constructions *CNX1E-G* or *CNX1G-E*, it is shown that the orientation of the domains CNX1E and CNX1G does not affect the functionality of the chimeric CNX1 proteins (Llamas et al., 2007). This finding suggests that the fact of having two genes *CNX1E* and *CNX1G*, or just one *CNX1* with the orientation E-G as in plants, or G-E as in mammals is irrelevant. In contrast to other Moco genes, *ABA3* (Abscisic acid deficient 3, in plants; Bittner et al., 2001) is not generally present in the analyzed algae (Table 1). ABA3 is required for functionality of the Mo-hydroxylases, Xanthine Dehydrogenase, and Aldehyde Oxidase, by the transfer of a sulfur atom to the Mo-center (sulfuration step). Thus, it will be interesting to understand what is the effect of this lack in the metabolism of those algae. Similarly, *MCP* encoding a Moco Carrier Protein (Ataya et al., 2003; Fischer et al., 2006) seems to be involved in an efficient mechanism of Moco storage and protection. MCP has a significant conservation with MBP (Moco Binding Protein) from plants (Kruse et al., 2010), but it is only present in Volvocales and not in the other algal genomes analyzed (Table 1).

Additionally, it is interesting to point out that the *CNX5* gene for Moco biosynthesis was differentially conserved in the studied algae. *CNX5* from *Chlamydomonas* and *Volvox* are closely related but markedly different from *CNX5* genes from the other algae, which show a significant conservation among them. The reasons for this diversification are not clear.

Concerning the ammonium incorporation to amino acids, the corresponding genes were analyzed in algae (Ghoshroy and Robertson, 2015). GS (*GLN*) genes are well-represented in Volvocales in a number of 3 or 4. However, their particular role and localization has only been studied in *Chlamydomonas* (Florencio and Vega, 1983a; Chen and Silflow, 1996; Vallon and Spalding, 2009). The four GS genes encode cytosolic glutamine synthetases of type 1 (GLN1, GLN4) and plastidic GS of type 2 (GLN2, GLN3). The two isoforms of GS2 are highly conserved, and their genes located head to head in the genome suggesting a recent event of gene duplication in both *Chlamydomonas* and *Volvox* (Chen and Silflow, 1996; Vallon and Spalding, 2009). In the species of *Ostreococcus* and *C. merolae*, the single *GLN* gene is more related to bacterial *GLN* than to plant-type *GLNs* present in other algae (Table 1). Genes for the plastidic NADH-GOGAT and Ferredoxin-GOGAT appear in Chlorophytes, however in Mamiellales, Rhodophytes, and Glaucophytes there appears only one (Ferredoxin-GOGAT).

## Regulation of nitrate assimilation

The regulation of nitrate acquisition is a complex process involving a network of proteins, many of them scarcely known. Majority of data available has been attained in *Chlamydomonas*. Below, we review some relevant regulatory aspects reported in microalgae.

In general, nitrate and ammonium have opposite effects over nitrate assimilation genes, which are referred to as positive (nitrate) and negative (ammonium) signals. Ammonium is the preferred N source due to its reduced state and energetically favorable assimilation. It is well-established that ammonium has a negative effect on nitrate assimilation at both transcriptional and posttranscriptional levels in *Chlamydomonas* (Fernandez and Galvan, 2008), especially in high CO_2_ conditions. NR gene (*NIA1*) expression, widely studied in many algal species, is induced in nitrate medium and strongly repressed in ammonium medium (Loppes et al., 1999; Cannons and Shiflett, 2001; Llamas et al., 2002; Imamura et al., 2010). Also nitrate transporters are expressed after ammonium depletion in *Micromonas* (McDonald et al., 2010). In *Chlamydomonas*, when both positive and negative signals are present together, repression appears as a quantitative process that is not strictly sensitive to the ammonium concentration but to the nitrate/ammonium (N/A) balance (Llamas et al., 2002; de Montaigu et al., 2011). A determined ammonium concentration is less repressive in conditions of high nitrate (mM) than in low nitrate (μM). Nevertheless, in medium without ammonium, *NIA1* is similarly induced by low and high nitrate concentrations. This pattern points out to a finely tuned regulation mediated by the N/A balance. How this balance regulates *NIA1* is scarcely known and a complex network of proteins and signal molecules are starting to be unraveled in *Chlamydomonas*. To understand these positive and negative signaling pathways, an insertional mutant collection of about 22,000 mutants was obtained and screened for aberrant sensing of nitrate or ammonium. The selection of these phenotypes was possible because the mutagenized strain bears a reporter gene that responds positively to nitrate and negatively to ammonium (*pNIA1ARS*, a chimeric gene: promoter of *NIA1* fused to the arylsulfatase gene; González-Ballester et al., 2005). For the ammonium insensitive phenotype (AI), a broad range of insensitiveness is shown depending on the mutant but none of them is completely insensitive to ammonium (de Montaigu et al., 2011). The numerous mutants isolated together with the absence of a mutant totally insensitive to ammonium highlight a regulation mechanism that probably involves several independent pathways. In contrast, the nitrate insensitive mutants (NI) reveal a master gene, *NIT2*, needed for nitrate assimilation genes expression (Camargo et al., 2007). In addition, a number of NI mutants with partial phenotypes are identified that also illustrates the complexity of nitrate regulation (Higuera et al., 2014).

### Positive regulation of the nitrate assimilation pathway

In photosynthetic organisms nitrate is not only an N source but also a signaling molecule (Crawford, 1995; Krapp et al., 2014). Nitrate induces expression of genes responsible for its assimilation. Proteins involved on nitrate sensing are scarcely known in microalgae. Several major transcription factors were identified in plants, fungi, yeast, and in some green and red algae. In *Chlamydomonas* the transcription factor NIT2 is essential for a proper upregulation of the main genes for nitrate assimilation (*NII1, NRT2.1, NRT2.2, NRT2.3, NAR2, NIA1, NAR1.1*, and *NAR1.6*; Quesada et al., 1993, 1994; Mariscal et al., 2006). Indeed, nit2 mutants are unable to grow in nitrate medium. The *NIT2* gene is also repressed by ammonium and induced in nitrate and N-free medium, highlighting that other yet unidentified transcription factors work upstream to *NIT2* (Camargo et al., 2007).

Structural characteristics of NIT2 show an RWP-RK domain (so called by the consensus sequence) within a leucine zipper domain; this motif may serve in dimerization and DNA binding (Camargo et al., 2007). This domain is also found in algal minus dominance (MID) proteins (Lin and Goodenough, 2007) and transcription factors from plants as the NIN-like proteins (NLP), some of them having a key role in regulating responses to nitrate availability (Konishi and Yanagisawa, 2013; Chardin et al., 2014). Actually, NLP7 seems to be a major player in the primary response to nitrate (Castaings et al., 2009; Marchive et al., 2013). Transcription factors containing RWP-RK domains are numerous in green algae but many of them have an unknown function. Proteins with significant homology to NIT2 have not been identified. However, it is noteworthy an RWP-RK transcription factor along the cluster of N metabolism related genes in *Micromonas pusilla*. Though clustering is not a strong evidence for its function, this gene is a good candidate to study in future experiments in this alga. Furthermore, other RWP-RK transcription factors that are upregulated in N starvation in *Chlamydomonas* (RWP1 and RWP10) have an expression that correlates with those of *LAO1* (encoding L-Amine Oxidase) and *CNX1E*, respectively (Gargouri et al., 2015). In addition to the RWP-RK domain, NIT2 shows a GAF domain at the N-terminal region of the protein. This motif could bind small molecules such as cGMP, NO or α-oxoglutarate (Little and Dixon, 2003; Rybalkin et al., 2003; Büsch et al., 2005). However, the function of this domain remains poorly studied, although it is known that it does not bind nitrate and is not involved in DNA binding (Camargo et al., 2007). Additionally, NIT2 possesses a glutamine-rich domain involved in protein-protein interactions, essential for NIT2 functionality, and a nuclear export sequence (NES) (Camargo et al., 2007). How NIT2 senses the nitrate signal for binding to different gene promoters is unknown. Nevertheless, a NES domain appears in the transcription factor NIRA involved in nitrate induction in *Aspergillus* (Narendja et al., 2002) and in NLP7 in *Arabidopsis* (Marchive et al., 2013). This NES sequence is involved in NIRA and NLP7 exportation to the cytosol in the absence of nitrate (Bernreiter et al., 2007; Marchive et al., 2013). In Chlamydomonas the NES domain of NIT2 is close to the GAF motif, thus binding of small molecules to GAF domain might expose or hide the NES domain facilitating its nuclear exportation. However, in *Chlamydomonas* NIT2 is important to activate nitrate-inducible genes and to upregulate genes in nitrate plus ammonium medium, a condition under which the nitrate assimilation genes are repressed, as the truncated hemoglobin *THB1* (Sanz-Luque et al., 2015). Therefore, a nuclear export would not be favorable in those conditions. Furthermore, NIT2 is also involved in the repression of genes downregulated by nitrate like the ammonium transporter *AMT1.1* (González-Ballester et al., 2004). It would be expected to find new proteins interacting with NIT2 to integrate the nitrate/ammonium balance and proportionate the specificity for a proper gene expression/regulation on each N condition.

In other green microalgae apart from *Chlamydomonas*, transcription factors involved in upregulating the nitrate assimilation genes are unknown. Nevertheless, in *Chlorella vulgaris* a unique GATA site is present in the promoter region of the NR gene (Dawson et al., 1996). This sequence works as a nitrate response element (NRE) and can bind specifically a *Neurospora crassa* NIT2 zinc-finger domain/glutathione S-transferase fusion protein. Thus, similar GATA-binding transcription factors could be responsible for the transcriptional regulation in this alga (Cannons and Shiflett, 2001). In the red alga *Cyanidioschyzon merolae* an R2R3-type MYB transcription factor, *MYB1*, has a key role in the nitrate upregulation of the main N assimilation genes (*NIA1, NII1, NRT2, AMT, GLN*). *MYB1*, as *CrNIT2*, is upregulated in nitrate and N-free medium and the transcription factor encoded binds directly to the promoter of all of these genes activating their expression (Imamura et al., 2009, 2010). This class of transcription factors is numerous in green algae but it has not been related to N metabolism. Nonetheless, two R2R3-type MYB type transcription factors directly bind to the promoter region of the glutamine synthetase gene in *Pinus sylvestris* (Gómez-Maldonado et al., 2004) and some MYB proteins are differentially regulated by the N source in *Arabidopsis* (Scheible, 2004). Thus, we could expect that MYB proteins would be also involved in N regulation in some algae.

In addition to NIT2, other regulatory protein NZF1 (nitrate zinc finger 1) involved in nitrate positive signaling was recently identified in *Chlamydomonas*. This is a tandem zinc finger protein CCCH-type that regulates *NIT2* expression and, indirectly, the nitrate assimilation genes (Higuera et al., 2014). NZF1 is proposed to have a role in *NIT2* polyadenylation. The nzf1 knockout mutant shows aberrant forms of polyadenylated *NIT2* mRNAs, which are not present in the wild type strain in nitrate medium. Thus, this nzf1 mutant has decreased levels of wild type *NIT2* transcript. Analogous transcription factors were not found in other algae, but a posttranscriptional regulation related to the mRNA stability is reported for the NR gene in *Chlorella vulgaris* (Cannons and Cannon, 2002). In this case, two different mRNAs are synthesized in response to short and long times of induction in nitrate. The long mRNA is less stable than the short version, which is induced in minutes when the repressor (ammonium or derivatives) is removed or when the inductor (nitrate) appears. Differences between these mRNAs are located in the 5′UTR. This supposes a strategy to perform a rapid adaptation to the changing environmental conditions.

Transcriptomics analysis showed other transcription factors that are upregulated in N starvation. One of them is NRR1, which encodes a SQUAMOSA promoter-binding protein, SBP, responds early to N starvation, and has a relevant role in TAG accumulation. NRR1 is hypothesized as a “master” transcriptional regulator for reprogramming gene expression of N metabolism during N deprivation (Schmollinger et al., 2014; Gargouri et al., 2015). However, more transcriptomic and proteomic studies in nitrate medium would be required to shed light about new regulatory factors.

### Negative regulation of the nitrate assimilation pathway

**Figure 3:**
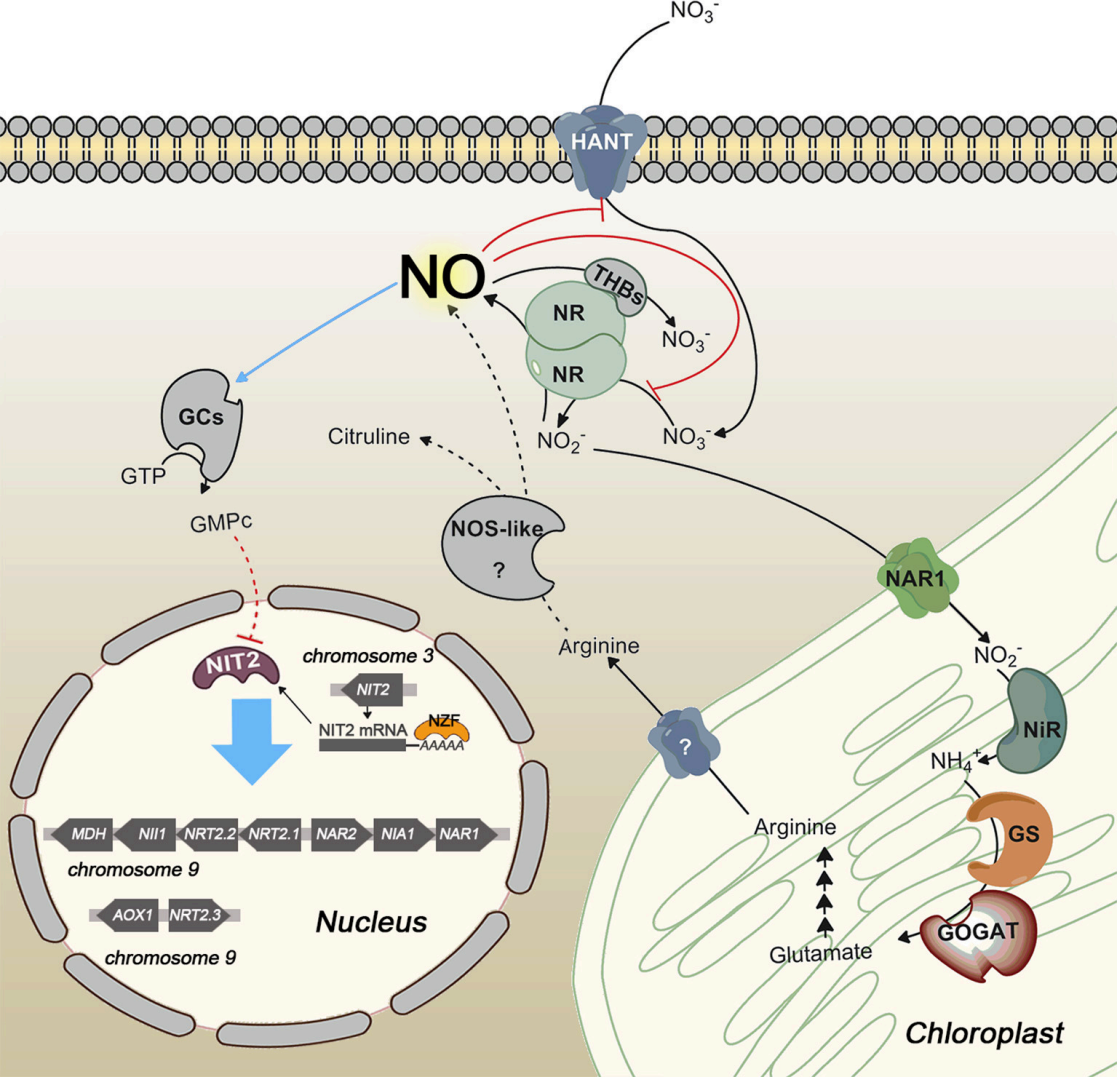
**Scheme of transcriptional and posttranslational negative regulation of nitrate assimilation in *Chlamydomonas***. Red and blue lines indicate inhibition and activation, respectively. Dashed lines represent hypothetic steps. NOS-like protein is an unidentified player that symbolizes the NOS activity reported in photosynthetic organisms. NO represses gene expression by activating a soluble guanylate cyclase (sGC) and inhibits NR activity and high affinity nitrate uptake. Two different sources of NO are represented, NR and a putative NOS enzyme. NO is scavenged by the complex NR/THB.

Nitrate assimilation is quickly blocked by ammonium addition. This is a common effect in the green algae studied. In fact, in higher plants, nitrate is essential to induce transcription of NR independently of ammonium. However, in algae removal of the repressor, or at least a shift in the balance N/A toward nitrate, is needed for induction (Cannons and Pendleton, 1994; de Montaigu et al., 2011). This repression is carried out at transcriptional (Quesada et al., 1993, 1998b; Berges, 1997; Loppes et al., 1999; Cannons and Shiflett, 2001; Imamura et al., 2010; de Montaigu et al., 2010) and posttranslational level (Florencio and Vega, 1983b; Franco et al., 1988; Galván et al., 1991, 1996). Some of the main players involved in transcriptional and posttranslational negative regulation of the nitrate assimilation pathway are briefly represented in Figure 3.

#### Transcriptional regulation

Genes encoding the NR and high affinity nitrate/nitrite/transporters are repressed by ammonium but the mechanisms and the players involved are starting to be uncovered in *Chlamydomonas*. In this alga 40 ammonium insensitive mutants were isolated (González-Ballester et al., 2005) and some of them studied. One of them, the cyg56 mutant, is affected in a soluble guanylate cyclase (sGC) important for the ammonium-dependent repression (de Montaigu et al., 2010). These enzymes produce cGMP from GTP and are activated when NO binds to their heme group, which is found in the HNOB domain (Heme NO Binding; Iyer et al., 2003; Poulos, 2006). CYG56 is a key enzyme in ammonium sensing and the cyg56 mutant is less sensitive to NO donors than wild type strain (704) and responds similarly to cGMP addition. Pharmacological approaches, using different NO and cGMP donors and GC and phosphodiesterase inhibitors, highlight cGMP and NO as important signal molecules, and sGC as a relevant player for the repression of genes that respond negatively to ammonium. Some genes repressed by the ${\text{NH}}_{4}^{+}$-NO-CYG56-cGMP mechanism are *NIA1, NRT2.1, AMT1.1*, and *AMT1.2*. Guanylate cyclase activity is poorly studied in photosynthetic organisms because in higher plants the canonical sGC does not exist. Nevertheless, cGMP is gaining a significant role in many processes in plants (Maathuis, 2006) and some proteins without homology to GCs like kinases and hormone receptors have shown GC activity (Ludidi and Gehring, 2003; Kwezi et al., 2007; Meier et al., 2010). Unlike plants, in the Chlorophyta phylum canonical sGC can be found. The *Chlamydomonas reinhardtii* and *Volvox carteri* genomes encode two vast families of 57 and 63 adenylyl/guanylyl cyclase domains, respectively, with several soluble GC among their members. This huge amount of cyclase domains highlights the importance of cyclic nucleotides in these algae, with a close phylogenetic relationship. Other chlorophytes used in this work have none or few canonical cyclase domains encoded in their genomes as *C. subellipsoidea* (1), *M pusilla* (1), *Micromonas* sp. RCC299 (1), *O. tauri* (2), and *O. lucimarinus* (1).

How cGMP regulates gene expression is unknown. A possibility is that cGMP binds to GAF domain of NIT2 (Camargo et al., 2007) and regulates its structure and binding to specific promoters, but deeper studies have to be performed to address and clarify this mechanism. Due to the similarity observed in the components of the nitrate assimilation pathway and similar patterns of regulation, we could expect that cGMP would have an important role in ammonium-mediated repression of nitrate assimilation genes in most of these algae. However, we propose that non-canonical GC, similar to those in plants, could have a relevant role in algae with few guanylate cyclase domains.

CYG56 expression is down-regulated in other AI mutants (de Montaigu et al., 2011). One of these strains (20.40) has a genome deletion affecting to nine putative genes; however, one of them was down-regulated in other AI mutants and is considered as a candidate for the AI phenotype. This gene codes a protein that shares homology with phosphofructokinases. In plants, these enzymes are regulated by 14-3-3 proteins (Kulma et al., 2004), like NR and GS (Finnemann and Schjoerring, 2000; MacKintosh and Meek, 2001), and may play a role in the cross-talk between nitrogen and carbon metabolisms. This mutant shows a high expression of *NIA1, NRT2.1*, and *AMT1.2* in nitrate medium, suggesting that this protein could be a general regulator rather than a specific player in ammonium sensing. Other relevant AI mutant (54.10) is affected in a gene, unidentified yet, that controls the expression of *CYG56* and *CDP1*, two genes that are independently involved in ammonium sensing. *CDP1* also shows higher expression in ammonium media and codes a cysteine-rich protein. CDP1 has a very low conservation in photosynthetic eukaryotes and do not show homology with known proteins. Nevertheless, a well-conserved CDP1 is found in *Volvox*. These mutants as well as new players and their relationships, albeit poorly characterized at molecular level, are bringing out new knowledge about the complex network working in this alga for a proper ammonium sensing.

Concerning regulatory mechanisms in other green algae, few data have been reported. In *Chlorella sorokiniana, NRT2.1* gene encoding nitrate transporter is induced by nitrate involving phosphorylation/dephosphorylation events (Koltermann et al., 2003), however, the players that mediate this regulation are unknown.

#### Posttranslational regulation

The ammonium-mediated negative effect over NR and nitrate transport was reported more than three decades ago. Ammonium addition, in cells induced with nitrate, inhibits in seconds the nitrate uptake (Florencio and Vega, 1982) and in minutes the NR activity (Franco et al., 1987). However, the mechanisms involved remain scarcely known. In plants, NR is regulated by phosphorylation of a serine residue, within a hinge region between the Moco and heme binding domains, followed by the binding of a 14-3-3 protein (MacKintosh and Meek, 2001; Lillo et al., 2004; Lambeck et al., 2012). Nevertheless, this serine residue is only conserved in higher plants and not in mosses nor algae (Medina-Andrés and Lira-Ruan, 2012). This points out that posttranslational regulation in microalgae should be different to that in plants and probably common with the most basal land plants, the mosses. Now, we are starting to understand how NR is post-translationally regulated in *Chlamydomonas*. NO not only triggers a cascade to repress transcription of the *NIA1* gene (see above, de Montaigu et al., 2010) but also partially inhibits the enzyme activity in a reversible way (Sanz-Luque et al., 2013). NO does not seem to interact directly with NR because the inhibitory effect appears when intact cells are treated but not when cell extracts or the *in vitro* recombinant NR are used. Recently, we have shown that the cytosolic truncated hemoglobin, THB1, has an important role in regulating NR activity (Sanz-Luque et al., 2015). This hemoglobin has NO dioxygenase activity (NOD) and takes up electrons from the FAD group of NR (diaphorase activity) to reduce its heme group. In the presence of NO, the reduced and oxygenated globin (THB1–${\text{Fe}}_{2}^{+}$–O_2_) catalyzes NO conversion to nitrate and decreases nitrate reduction by removing the reducing power available for NR. THB1 could also work with other reductase enzymes and probably mediate the control of other processes in the cell. However, due to its N-dependent transcriptional regulation we suggest that NR might be one of its most relevant partners. Curiously, two microalgae belonging to the class Raphidophyceae have the NR gene fused to a truncated hemoglobin (Stewart and Coyne, 2011). Authors hypothesize that this fusion confers a significant advantage to use the NO produced during bloom conditions as N source. These algae would be able to efficiently convert NO into nitrate by the NOD activity and subsequently to carry out the nitrate reduction.

In addition, other phosphorylatable serine residue, located in the hinge region 2 of NR between the FAD and heme domains, is highly conserved in both higher plants and algae. In *Arabidopsis*, MAPK6 phosphorylates this serine in NIA2 increasing the NR activity and NO production (Wang et al., 2010, 2014b).

Additionally, NO inhibits in a fast and reversible way the high affinity nitrate/nitrite transport (Sanz-Luque et al., 2013). Nitrate and nitrite uptakes of nitrate-induced cells are quickly halted after NO addition at low concentration, and normal rates of uptake recover after a few minutes, when NO disappears. In plants, two ways of regulation are described for nitrate transporters. NRT1.1, which is regulated by phosphorylation (Tsay et al., 2007; Ho et al., 2009) and some NRT2, which require association with the NAR2 protein to be active (Quesada et al., 1994; Zhou et al., 2000; Okamoto et al., 2006). Even so, the mechanisms and the players that connect the signal sensing with the posttranslational modification are unclear. NO could trigger cascades to phosphorylate or to modify directly the transporter or the accessory protein by S-nitrosylation.

Unlike NR and high affinity nitrate/nitrite transporters, NiR does not seem to be regulated by NO (Sanz-Luque et al., 2013). Regulatory mechanisms are unknown for NiR, although its activity depends on the reduced ferredoxin pool, which can be affected if photosynthesis decreases. That makes sense from a physiological point of view because this coordination avoids N loss, due to nitrite excretion, and possible toxic effects derived from nitrite accumulation. Under low photosynthetic activity rates NiR activity is low (Jin et al., 1998), resulting in the uncoupling of NR and NiR activities. Then nitrite production by NR can be higher than nitrite reduction to ammonium by NiR. Under this condition cells do not store the overproduced nitrite, which is excreted as an emergency strategy (Navarro et al., 2000). This excretion would entail loss of N and reducing power used for nitrate reduction. However, *Chlamydomonas* has a mechanism, which avoids such a disadvantage. In this alga one of the main NO sources is nitrite (Sakihama et al., 2002), and so, when nitrite accumulates, NR is able to synthetize NO with the subsequently inhibition of nitrate uptake and reduction until NR and NiR activities are coupled again (Figure 3).

The ammonium-dependent transcriptional and posttranslational regulation could be directly mediated by ammonium itself or by an ammonium derivative metabolite such as glutamine. Indeed, *CrAMT1.1* transcription (González-Ballester et al., 2004) or nitrite uptake (Galván et al., 1991), which are repressed and inhibited, respectively, by ammonium, are partially released of ammonium negative signaling by methione sulfoximine (MSX), an specific glutamine synthetase inhibitor. Glutamine has been described as an important N status reporter in many bacteria but in photosynthetic eukaryotes glutamine sensing is poorly understood. In many organisms glutamine is sensed by the PII protein, which is one of the most widespread signaling proteins in nature. These proteins are present in different kingdoms of life from bacteria to plants (Chellamuthu et al., 2013) and recently are reported in green algae as *Chlamydomonas* (Ermilova et al., 2013), *Chlorella* (Minaeva and Ermilova, 2015), and *Micromonas* (McDonald et al., 2010). Nevertheless, PII proteins are not conserved in all photosynthetic algae, genome analysis failed to reveal any obvious PII homologs in the red alga *Cyanidioschyzon merolae* and diatoms (Uhrig et al., 2009).

PII proteins are responsible for the integration of N and carbon metabolisms as well as of the cellular energy status by regulating transcription factors, membrane transporters and metabolic enzymes (Uhrig et al., 2009). In bacteria there exist at least four recognized PII proteins and several targets identified. However, in most cyanobacteria, microalgae and higher plants the number of PII proteins seems to be reduced to one and its functionality is barely known (Ninfa and Jiang, 2005; Ermilova et al., 2013). In *Chlamydomonas*, like in the other photosynthetic organisms, PII protein (GLB1) is localized in the chloroplast (Ermilova et al., 2013) and activates N-acetyl-L-glutamate kinase (NAGK), a key enzyme of arginine biosynthesis (Chellamuthu et al., 2014). CrGLB1 binds glutamine at millimolar concentration and then interacts and activates NAGK. If α-oxoglutarate concentrations are high the complex is inhibited and NAGK activity decreases. Therefore, when N/C balance is shifted to N the arginine biosynthesis is enhanced. Thus, in these conditions arginine could be used as N-storage molecule or as NO precursor by a putative NO Synthase activity (NOS). Curiously, *Arabidopsis* PII mutants show higher nitrite uptake to the chloroplast than wt (Ferrario-Méry et al., 2008). Nevertheless, the PII role in algae and plants is starting to be unraveled. Recently, RNA-seq data have shown that *CrGLB1* is overexpressed in N starvation (Schmollinger et al., 2014) but its role in this condition is unknown. Additional studies will be necessary to elucidate the PII participation in the regulatory processes of the N assimilation pathway.

### NO metabolism

In the last years NO has emerged as an important signal molecule, which regulates many processes in plants and algae. A huge amount of publications have related it with essential roles in plant growth, development, defense (Delledonne et al., 1998; Corpas et al., 2004; He et al., 2004; Fernández-Marcos et al., 2011) and nitrate metabolism in plants and algae (Du et al., 2008; Jin et al., 2009; de Montaigu et al., 2010; Rosales et al., 2011; Sanz-Luque et al., 2013). Nevertheless, and despite the fact of being an important signal molecule involved in a vast number of processes, our knowledge about its synthesis and turnover in photosynthetic organisms is poor. Related to the synthesis, two different pathways are possible, the oxidative one from arginine, polyamines, or hydroxylamines, and the reductive one from nitrite (Wilson et al., 2008; Moreau et al., 2010; Gupta et al., 2011a). In photosynthetic organisms the main NO source described is NR that reduces nitrite to NO *in vivo* and *in vitro* using NAD(P)H as electron donor. This activity has been described in both plants (Yamasaki and Sakihama, 2000; Rockel et al., 2002) and algae (Mallick et al., 2000; Sakihama et al., 2002). Although the molecular basis of this enzymatic activity is not well-characterized, there exists a growing number of physiological processes regulated by a NR-dependent NO synthesis in plants and algae (Zhao et al., 2009; Hao et al., 2010; Lozano-Juste and León, 2010; Horchani et al., 2011; Wei et al., 2014; Sun et al., 2015). The NO synthesis by NR has been estimated as 1% of the nitrate reducing capacity (Rockel et al., 2002). Thus, due to the higher affinity for nitrate than for nitrite, the NO production would require a low nitrate/nitrite balance. Indeed, experiments without nitrate are used to measure this activity (Sakihama et al., 2002), and when nitrate is added a significant delay is observed (Yamasaki and Sakihama, 2000) supporting the importance of a shifted nitrate/nitrite balance in NO synthesis by NR. As described above, NR-dependent NO synthesis would inhibit nitrate uptake and reduction with the aim of avoiding the accumulation of toxic nitrite and/or its excretion when NR and NiR are uncoupled.

In addition to NR, other reductive pathways for NO synthesis in photosynthetic organisms were described. Recently, a NR-independent NO source has been reported in *Chlamydomonas*, where nit2 mutants, with no expression of NR, showed NO increase in N-free medium after 20 h (Wei et al., 2014). Several of the alternative NO sources in plants are a plasma membrane-bound nitrite:NO reductase (Stöhr et al., 2001), which has been mainly studied in tobacco, and mitochondrial (Tischner et al., 2004; Gupta and Igamberdiev, 2011) and plastidic (Jasid et al., 2006) nitrite reduction. This reduction could be mediated by molybdoenzymes, as Amidoxime reducing component, of unknown function (Yang et al., 2015), Xanthine oxidoreductase in plants and animals (del Río et al., 2004; Gupta et al., 2011a) or Aldehyde oxidase (Li et al., 2009) and Sulfite oxidase (Wang et al., 2014a) reported only in animals albeit they could own a similar activity in plants. Finally, chemical apoplastic nitrite reduction to NO at acidic pHs has also been described (Bethke et al., 2004).

The oxidative production of NO in plant-like organisms is poorly known at molecular level. Arginine-dependent NO synthesis is mediated by NOS, but no protein with homology to animal and bacterial NOS has been found in plants. Even so, an arginine-dependent NO production has been physiologically described (Corpas et al., 2006, 2009; Moreau et al., 2010). Surprisingly, only two photosynthetic organisms bearing genes that encode animal-type NOS enzymes in their genomes have been reported up to date, the small algae *Ostreococcus tauri* and *Osteococcus lucimanirus* (Foresi et al., 2010, 2015). The recently released genome of *Bathycoccus prasinos* (Moreau et al., 2012), other small alga from the Mamiellales clade of prasinophyceae as *Ostreococcus*, also has a gene encoding a NOS enzyme with 62% of similarity to *O. tauri* NOS (Kumar et al., 2015). Curiously, these primitive algae are species with small genomes and high horizontal gene transfer. In fact, a phylogenetic study suggests that around 5% of the *Bathycoccus prasinos* genome was originated by horizontal gene transfer (Moreau et al., 2012). This may explain why *Bathycoccus* and *Ostreococcus* are exceptional algae having genes that encode animal-type NOS enzymes.

Inhibition of nitrate reduction and uptake when nitrite concentration increases can be mediated by NO produced in a NR-dependent way; however, the NO source that performs the ammonium repression seems to depend on an arginine-dependent process in *Chlamydomonas*. Experiments with L-NAME (*N*_ω_-Nitro-L-arginine methyl ester), an analog of arginine used as inhibitor of NOS, show a partial release of the ammonium repression of *NIA1* and other genes induced by nitrate and repressed by ammonium (de Montaigu et al., 2010). Nevertheless, a role of NR in ammonium repression cannot be discarded.

Other important aspect of NO metabolism is its fast removal to avoid unspecific reactions producing toxic effects. Hemoglobins (Hb) have arisen as relevant proteins involved in NO scavenging. In plants, non-symbiotic Hbs have shown to have this role (Perazzolli et al., 2004; Gupta et al., 2011b). In algae, as described above, truncated Hbs are relevant players in this task. In *Chlamydomonas* a vast family of 12 truncated hemoglobins are found (Hemschemeier et al., 2013) and some of them were related to different physiological processes. Two of them THB1 and THB2 are differentially expressed by the N source, showing high transcription levels in nitrate and nitrate plus ammonium, and under the control of NIT2 (Johnson et al., 2014; Sanz-Luque et al., 2015). However, opposite responses to NO are observed for *THB1*, which is upregulated, and *THB2*, which is downregulated. In addition, as stated above, THB1 has NOD activity working with NR and using reducing power from NAD(P)H. Other recently studied truncated hemoglobins in *Chlamydomonas* are THB8, which is required for hypoxic survival in a process involving NO (Hemschemeier et al., 2013), or THB7, THB10, and THB11 that are overexpressed in the dark (Huwald et al., 2015). Some of these proteins (THB1-2-4-7-10-11) are biochemically characterized but our knowledge about their functions is poor (Ciaccio et al., 2015; Huwald et al., 2015; Rice et al., 2015). Therefore, in the last years this microalga has become an interesting organism to study the NO metabolism and functionality of these proteins. Truncated hemoglobins have also been identified in other *Chlamydomonas* species as well as in *Volvox carteri, Chlorella* sp. NC64A and *Micromonas pusilla* (Vázquez-Limón et al., 2012; Hemschemeier et al., 2013).

Additionally, other reactions that mediate NO scavenging are: spontaneous oxidation to nitrite; reaction with glutathione to store it as nitrosoglutathione (GSNO), which is the main NO reservoir *in vivo* in photosynthetic organisms (Wang, 2006); reaction with other ROS as superoxide anion to produce peroxynitrite, which works as a signal molecule in protein modification and, finally, reaction with metals as iron of heme groups (Moreau et al., 2010; Gupta et al., 2011a; Mur et al., 2013). Nevertheless, these mechanisms are less studied in algae.

### Future perspectives/conclusions

The nitrate assimilation pathway is a relative simple process conserved in the majority of microalgae. This conservation highlights the importance of this pathway in the central metabolism of these organisms. This pathway owns an uptake step to incorporate nitrate into the cell, a first NR-mediated reduction followed by nitrite incorporation to chloroplasts and a second reduction step mediated by NiR. Finally N is incorporated into carbon skeletons by GS/GOGAT cycle. Additionally, the key enzyme for nitrate assimilation (NR) harbors molybdenum cofactor, and subsequently the genes responsible for Moco biosynthesis are also highly conserved in most studied algae. In spite of the low structural complexity of this pathway its regulation is intricate. A complex protein network controlling nitrate assimilation is starting to be unraveled in *Chlamydomonas*. However, although structural genes for nitrate assimilation are well-conserved, being clustered in most algae, those involved in regulation show a lower conservation degree. Even so, a central role of NO and cGMP occurs in *Chlamydomonas*, and so the implication of these signal molecules in the regulation of other algae must be addressed. The higher diversification in the regulatory processes would allow a specific adaptation of each alga to its particular environment. At structural level, main differences are found in the number of transporters. *Chlamydomonas* is an alga with a high number of them, but other, algae with small genome sizes have transporter families with only one member. Probably, this would be a good reason to expect also a more complex regulation in *Chlamydomonas*. These features among others contribute to the high adaptability observed for this organism and have converted *Chlamydomonas* in a good model alga for basic science and biotechnological use. Nevertheless, future studies should improve our knowledge about the whole system of nutrient acquisition in algae, addressing not only assimilation of different N forms and its regulation but also their relationships with carbon, sulfur and phosphorus metabolisms. This understanding will allow us to develop many applications with algae and discover the real potential of these organisms on applied sciences.

## Funding

This work was funded by MINECO (Ministerio de Economía y Competitividad, Spain, Grant no. BFU2011-29338) with support of European Fondo Europeo de Desarrollo Regional (FEDER) program, Junta de Andalucía (P08-CVI-04157, BIO-128, and BIO-286), and Plan Propio de la Universidad de Córdoba.

### Conflict of interest statement

The authors declare that the research was conducted in the absence of any commercial or financial relationships that could be construed as a potential conflict of interest.

## Abbreviations

Fd_red_: Reduced ferredoxin
GOGAT: Glutamine oxoglutarate amino transferase or glutamate synthase
GS: Glutamine Synthetase
HATS: High affinity transporters
Moco: Molybdenum cofactor
N: Nitrogen
NiR: Nitrite Reductase
NO: Nitric Oxide
NOS: Nitric oxide synthase
NR: Nitrate Reductase
sGC: soluble guanylate cyclase.
