# Supplementary material for: Understanding nitrate assimilation and its regulation in microalgae
Source: Front Plant Sci. 2015 Oct 26;6:899. doi: 10.3389/fpls.2015.00899 (PMC4620153; doi:10.3389/fpls.2015.00899)
Supplement: Supplementary file 1 [file Table1.DOCX]

|  | *C. reinhardtii* | *V. carteri* | *C. subellipsoidea* | *M. pusilla* | *Micromonas RCC299* | *Chlorella NC64A* | *O. lucimarinus* | *Ostreococcus RCC809* | *O. tauri* | *B. prasinos* | *C. merolae* |
| --- | --- | --- | --- | --- | --- | --- | --- | --- | --- | --- | --- |
|  | *Chlorophytes* | | | | | | | | | | *Rhodophytes* |
| *NRT1* | Cre04.g224700 | Vocar20011804 | 19259  48785 | NF | 62932 | 144528  23105 | 50811 | 59393 | 2706 | Bathy13g01850 | CMO266C |
| *NRT2* | **Cre09.g410850**  **Cre09.g410800**  Cre09.g396000  Cre03.g150101  Cre03.g150151  Cre02.g110800 | **Vocar2004706**  **Vocar20004282**  Vocar20004618 | 28993 | **49583** | **104706**  105997  **107297** | **26630**  **138674** | **26062** | **52318** | **24168** | **Bathy02g01490** | **CMG018C** |
| *NAR1* | **192094**  Cre06.g309000  Cre04.g217915  Cre07.g335600  Cre12.g54125  Cre01.g012050 | **Vocar20004402**  Vocar20013042  Vocar20011948  Vocar20005659  Vocar20006291 | 20872  46302 | **70731** | **113973** | 34412  25301  53335 | **25116** | **24496** | **19784** | **Bathy02g01520** | NF |
| *NAR2* | **Cre09.g410900** | **Vocar20004699** | 47957 | **70904** | **104705**  **112726** | **138676** | **17012** | **70567** | **24167** | **Bathy02g01500** | NF |
| *NR* | **Cre09.g410950** | **Vocar20004278** | **37154**  29226 | **39565** | **113975** | **56304** | **37938** | **37605** | **19576** | **Bathy02g01530** | **CMG019C** |
| *NiR* | **Cre09.g410750** | **Vocar2004474** | **29833** | **70828** | **113259** | **26644** | **50765** | **59118** | **26396** | **Bathy02g01510** | CMJ117C  **CMG021C** |
| *GLN* | Cre02.g113200  Cre12.g530650  Cre12.g530600  Cre03.g207250 | Vocar20009413m  Vocar20014904m  Vocar20014850m  Vocar20005560m | 23194  23517  30043 | 4228 | 58286 | 56005  143431 | 39960 | 89446 | 15060 | Bathy01g02790 | CMI233C |
| *GSN/GSF* | Cre13.g592200  Cre12.g514050 | Vocar20013497m  Vocar20014037m | 53183  22625 | 57115  70244 | 105473  63658 | 33619  142154 | 51965 | 61091 | 29431 | Bathy12g01040 | CMV060C |
| *MCP* | Cre09.g390050 | Vocar20003720 | NF | NF | NF | NF | NF | NF | NF | NF | NF |
| *MOT1* | Cre04.g214050 | NF | 3089 | NF | 101424 | 138543 | **50753** | **94245** | **3075** | Bathy01g03650 | NF |
| *MOT2* | Cre13.g580150  Cre16.g695500 | Vocar20009935  Vocar20006177 | 47597  48086 | 52340  12882 | 63950  83429 | 7908  8090 | 30525  3784 | 56358  57792 | 8261  2827 | Bathy09g01790  Bathy07g02160 | CMO210C |
| *CNX2* | Cre13.g602901 | Vocar20013875m | 24726 | **70905** | 83859 | 49033 | **50634** | **37660** | **19513** | Bathy08g03180 | CMR288C |
| *CNX3* | Cre08.g361050 | Vocar20013038m | 37164 | 50687 | 113905 | 138003 | 119780  14397 | 25140 | 8221 | Bathy14g02080 | CMN213C |
| *CNX5* | Cre09.g389050 | Vocar20010865m | 36641 | 51160 | 113992 | 22673 | **38170** | **68677** | **19906** | Bathy13g02860 | CMM289C |
| *CNX6* | Cre07.g322250 | Vocar20003232m | 38113 | 53761 | 85418 | 17589 | 37690 | 42038 | 22035 | Bathy06g00080 | CME167C |
| *CNX7* | Cre08.g382545 | Vocar20010994m | 12278 | 52674 | 64811 | 142748 | 94198 | 28190 | 30576 | Bathy09g02710 | - |
| *CNX1G* | Cre06.g282150 | Vocar20002809m | 18464 | 60392 | 62280 | 137862 | 8506 | 91876 | 33158 | Bathy10g00300 | CMS449C |
|  |  |  |  |  |  |  |  |  |  | Bathy05g00280 |  |
| *CNX1E* | Cre10.g451400 | Vocar20000663m | 47932 | 44375 | 79735 | 58800 | 40817 |  |  |  | CMB143C |
| *ABA3* | Cre03.g152000 | Vocar20000623m | 19457 | NF | 64112 | 54300 | NF | NF | NF | Bathy10g01940 | NF |

**Table S1. Accession numbers of Nitrate and Molybdenum assimilatory proteins in microalgae.** We have used the specific genome databases for each organism. Some proteins were already identified and annotated and others have been found using BlastP and TBlastN. *Chlamydomonas reinhardtii*: Phytozome 10.2 (http://phytozome.jgi.doe.gov/ pz/portal.html), for NAR1(192094) we used JGI v4 (http://genome.jgi-psf.org/). *Volvox carteri, Coccomyxa subellipsoidea, Micromonas pusilla, Micromonas RCC299* and *Ostreococcus lucimarinus*: Phytozome 10.2. *Chlorella NC64A, Ostreococcus RCC809* and *Ostreococcus tauri:* JGI. *Bathycoccus prasinos: G*enome.jp (www.genome.jp/kegg-bin/show_organism?org=bpg). *Cyanidioschyzon merolae:* *Cyanidioschyzon merolae* Genome Project (http://merolae.biol.s.u-tokyo.ac.jp/blast/blast.html). Identified proteins are NRT1, NRT2 and NAR1: nitrate/nitrite transporters, NAR2: a necessary protein for a two-component high affinity nitrate transport, NR: Nitrate Reductase, NiR: Nitrite Reductase, GLN: Glutamine synthetase, GSN/GSF: Glutamate synthase, MCP: a Moco Carrier Protein, MOT1 and MOT2: Molybdate Transporters, Cnx2, Cnx3, Cnx5, Cnx6, Cnx7, Cnx1G, Cnx1E and ABA3: Proteins of Molybdenum biosynthesis. (NF) indicates that proteins were not found in that organism. Accession numbers of proteins from *Galdieria Sulphuraria* and *Cyanophora paradoxa* are not annotated in this table because in their genome database (http://genomics.msu.edu/cgi-bin/galdieria/blast.cgi and http://cyanophora.rutgers.edu/cyanophora/blast.php, respectively) the ID for these proteins there not exist, however proteins can be found by Blast. Highlighted in bold are those proteins encoded by genes that are grouped in cluster for each organism.
